# Do Local Religious Beliefs Affect Firms’ Earnings Management Practice? Evidence From the United States

**DOI:** 10.3389/fpsyg.2022.883596

**Published:** 2022-06-13

**Authors:** Hongying Geng, Min Hua, Li Sun, Chao Yan

**Affiliations:** ^1^Institute of Economics and Management, Harbin University, Harbin, China; ^2^Faculty of Professional Finance and Accountancy, Shanghai Business School, Shanghai, China; ^3^Research Center of Finance, Shanghai Business School, Shanghai, China; ^4^Center for Quantitative Economics, Jilin University, Changchun, China

**Keywords:** religion, real earnings management, corporate governance, social cultural, social norms

## Abstract

This paper investigates whether local religious beliefs have a significant impact on the practice of earnings management. We extend the existing literature on the role of firm characteristics in mitigating earnings management by showing that local religious beliefs significantly impact the practice of earnings management. Specifically, exploring firms located in the U.S. counties that vary from 2000 through 2010, we document the negative relationship between religiosity and earnings management using multivariate regression analysis. Our results show that firms in counties with strong religious social norms are less likely to engage in earnings management. Furthermore, we attempt to mitigate endogeneity concerns by employing a modified Difference-Differences model and Propensity score matching methods. We find that the negative effects of religion on earnings management still hold. Overall, these findings emphasize the empirical relevance of the association between the local social norms and earnings manipulations.

## Introduction

Earnings management has received considerable attention from regulators and the popular press. Managers have incentives to manipulate earnings by choosing reporting methods and estimates that do not fully reflect firms’ underlying economics for their own benefits (Healy and Wahlen, 1999). Therefore, earnings manipulation activities are considered opportunistic and unethical behavior by masking and concealing the actual information that investors and shareholders are supposed to know (Loomis, 1999). Consequently, the bulk of studies implies that earnings management negatively affects firm performance and long-term shareholder wealth (Klein, 2002; Chi and Gupta, 2009). In an effort to mitigate the earnings management, a recent trend is to offer insight on internal corporate governance mechanisms that can solve this problematic issue, including the Board Structures and compositions (Park and Shin, 2004); ownership structure (Bao and Lewellyn, 2017) and managers’ Compensation incentive (Laux and Laux, 2009). In this paper, we aim to extend this line of studies by investigating whether the local social norm is a key driver that can reduce the extent of earnings management.

Religion has long been considered an essential part of economic thought (Smith, 1776; Anderson, 1988). Prior research has revisited the analysis of religions and extensively examined the pivotal influences of religion on personal risk propensity (Miller and Hoffmann, 1995), economics attitudes (Guiso et al., 2003), and individuals’ behaviors (Kennedy and Lawton, 1998). Moreover, recent studies further show that local religious beliefs profoundly affect corporate decision-making. Hilary and Hui (2009) showed that firms located higher in religious adherents have more conservative investment strategies. McGuire et al. (2012) find that firms in religious areas are negatively associated with financial reporting irregularity. However, little is known about whether earnings management in the firm can be modified by local religiosity. Our work thereby attempts to complement these studies by examining the link between religion and earnings management by focusing on counties in the U.S.

Our paper contributes to the existing literature from three perspectives. First, our paper enriches the studies of religion by paying attention to its effect on earnings management. There is plentiful research that has identified religion as a key driver of economic behavior at both macro and micro level for a long time after Weber’s (1905) seminal work. For example, Barro and McCleary (2003) reveal that religion is associated with economic growth. Guiso et al. (2003) imply that religious beliefs averagely show “good” economic attitudes. For the micro-level effects, previous studies demonstrate that local religiosity imposes prominent influences on organizational behaviors, such as decision-making (Giannetti and Yafeh, 2012), risk-taking (Hilary and Hui, 2009), and questionable activities (Boone et al., 2013; Callen and Fang, 2015). However, to the best of our knowledge, limited studies explore the relationship between religiosity and earnings management. Our findings extend this line of research by suggesting that firms located in counties with higher levels of religiosity can mitigate the practice of earnings management. Second, this study furthers the literature on the determinants of firms’ earnings management activities. Previous studies focus on how corporate governance affects earnings management. For example, Liu and Lu (2007) document that higher corporate governance levels have lower levels of earnings management by examining Chinese listed companies. Klein (2002) implies that audit committee and board characteristics are vital factors that affect earnings management. Moreover, social norms such as religions have also proven to significantly influence organizational behaviors profoundly and widely in recent decades. Hilary and Hui (2009) reveal that firms located at higher levels of religiosity show lower degrees of risk exposure. McGuire et al. (2012) suggest that firms in regions with strong religious social norms experience lower incidences of financial reporting irregularities. Our work attributes to this stream of studies by showing that local religiosity is a crucial determinant of firms’ earnings management. Third, prior studies indicate that implementing the Sarbanes-Oxley Act (SOX) of 2002 significantly affects earnings management activities (Cohen et al., 2008; Hossain et al., 2011; Hsu and Huang, 2020). We thereby carried out a natural experiment by using the regulation of the Sarbanes-Oxley Act (SOX) of 2002 as an exogenous shock of tenure-weighted religion and listing the requirements by NASDAQ and NYSE for firms to have a majority of independent directors. To our best knowledge, we are the first to use the modified Difference-in-Differences model to estimate Religion’s “clean” effect on earnings management in the company. Our works, therefore, attribute to the literature that investigates the relationship between social norms and the practice of earnings manipulations.

In doing so, we use 20,862 firm-year observations for 3,810 unique firms from 2000 through 2010 to examine the influences of religion on earnings management. Our main variable Religion is calculated as the number of religious adherents in the county to the total population in the county as reported by ARDA. Following previous studies, we also linearly interpolate the data to obtain the values for missing years (1972–1979, 1981–1989, and 1991–1999) (Alesina and La Ferrara, 2000; Hilary and Hui, 2009). Our results indicate that firms located in a county with high levels of religiosity reduce the extent of earnings management. However, our findings could be biased due to endogeneity issues. For example, our main results could be spurious due to unobservable characteristics of tenure-weighted co-option. We alleviate this concern by using the difference-in-differences (DiD) approach. Specifically, following the previous paper, we carry out a natural experiment by using the regulation of the Sarbanes-Oxley Act (SOX) of 2002 as an endogenous shock of tenure-weighted religion and listing the requirements by NASDAQ and NYSE for firms to have a majority of independent directors (Coles et al., 2014). Another concern is self-selection bias, as managers make corporate decisions according to their preferences. To address this issue, we adopt a propensity-score-matching procedure suggested by prior literature (Rosenbaum and Rubin, 1983; Lin et al., 2018). Overall, our identification strategy provides evidence that religion negatively affects earnings management.

The paper is organized as follows: Section 2 describes how our study relates to the existing literature. Section 3 describes our data and methodology. Section 4 presents the empirical results. The final section summarizes our findings.

## Literature Review and Hypothesis Development

### Earnings Management Literature Review

Earnings management is a prevalent topic that researchers have investigated for a long time. One of the prominent arguments that define earning management is that it occurs when firms’ managers use their judgment in financial reporting and structuring transactions to alter financial reports to mislead some stakeholders (Healy and Wahlen, 1999). Aggressive earnings management is considered opportunistic and unethical behavior as it window dress the information that investors and shareholders are supposed to know (Loomis, 1999). Consequently, this behavior destroyed the trust between shareholders and companies (Levitt, 1998). Plenty of research, therefore, provides empirical evidence to highlight the consequences of earnings management while exploring the determinants that can alleviate the phenomenon of earnings manipulations.

From the opportunistic perspective, earnings manipulation activities decrease the reliability of financial reporting and generate information asymmetry problems between inside and outside investors, which negatively affect stock market liquidity (Hunjra et al., 2020). Besides, several other studies demonstrate that earnings management has a negative influence on firm performance (Teoh et al., 1998; Louis, 2004; Cormier and Martinez, 2006), increasing the cost of capital in the company (Kim and Sohn, 2013) and positively affect corporate credit risk after examining Pakistani manufacturing listed firms (Hunjra et al., 2022). Evidence indicates the severe consequences of aggressive earnings manipulation activities in firms. In the light of studies that explore the key factors that can eliminate earnings management, this aspect has also been extensively investigated. Liu and Lu (2007) focus on corporate governance and indicate that the levels of corporate governance significantly affect earnings management in China. Klein (2002) implies that the independence of audit committee and board directors are negatively related to earnings management by the firms. This paper extends this line of studies by showing that local religiosity level does matter in firm earnings management.

### Religion and Related Literature

There is strong theoretical support for the effects of religiousness on business ethics (Fort, 1997). Religion, which serves as an important social institution, plays an integrative role in societies and individuals within them (Huffman, 1988). It exercises control over individuals’ recognition of the social value and their behaviors (Kennedy and Lawton, 1998). Therefore, a bulk of studies explore the impacts of religions, and results of plentiful previous empirical research identify the crucial role of religion on economic behavior at both macro and micro levels for a long time after Weber’s (1905) seminal work. For example, Barro and McCleary (2003) reveal that religion is associated with economic growth. Guiso et al. (2003) imply that religious beliefs averagely show “good” economic attitudes. Nowadays, religions are well-developed and typically and extensively being investigated using micro-level data.

The existing literature offers several aspects of the role of religions in the corporate finance area. For corporate decision-making, Hilary and Hui (2009) explore the relationship between corporate culture and organizational behavior in America. They find that the levels of religiosity in firms located significantly affect the degrees of risk exposure in the company. Elnahas et al. (2017) display the possible effect of religion on M&As by showing that Islamic law can mitigate some prohibited conditions in conventional earnout contracts that exist in M&As. In addition, the most recent study indicates that firms bundled with religiosity outperform their counterparts when examining the effects of CSR activities on firm performance (Hunjra et al., 2021). Furthermore, religions are also related to some questionable activities. Boone et al. (2013) examine the religiosity level of firms’ location as a key determinant of aggressive tax avoidance strategies. Similarly, several studies suggest that religion significantly influences the quality of financial reporting. McGuire et al. (2012) demonstrate that firms headquartered at a higher level of religious social norms are less likely to experience financial reporting irregularities. Dyreng et al. (2012) show a significant and negative relationship between religious social norms and financial reporting aggressiveness. Our work explores the role of religions on firms’ earnings management activities.

### Hypothesis Development

As religion help individuals shape their beliefs and behaviors (Kennedy and Lawton, 1998), previous evidence shows that religious beliefs are less likely to be involved in unethical activities as unacceptable practices (Conroy and Emerson, 2004). This is due to the reason that religion provides adherents guidelines that help identify ethical and unethical experiences (Weaver and Agle, 2002). As a result, to some extent, religions are reasonable to affect corporate behaviors. Prior studies have provided evidence that firms located in a high religiosity area can alleviate the prohibited unethical or questionable corporate activities (Dyreng et al., 2012; Boone et al., 2013; Hofmann and Schwaiger, 2020). Furthermore, empirical studies provide evidence that local religiosity can affect the decision makings of local legislations, local government policies and business-related regulations (e.g., Bhalotra et al., 2014; Chen et al., 2016), which in turn can affect corporate behaviors directly. Building on those arguments and empirical results, we are aware that local religious culture is reasonable to affect the firms’ operations in the area. We hypothesize that high levels of religious adherence surrounding a firm’s location will reduce firms’ management earnings activities.

## Data and Methodology

### Sample Selection

Following Hilary and Hui (2009), we calculate Religion as the number of religious adherents in the county to the total population in the county as reported by ARDA.^1^ Following previous studies (e.g., Alesina and La Ferrara, 2000; Hilary and Hui, 2009), we linearly interpolate the data to obtain the values for missing years (1972–1979, 1981–1989, and 1991–1999). Our sample consists of the universe of Compustat firms over 2000–2010. Data on institutional ownership were obtained from 13F Thomson Reuter. Next, we merged these observations with stock-level data from the Center for Research in Security Prices (CRSP) and analyst data from the Institutional Brokers’ Estimate System (I/B/E/S). Our final sample included 20,862 firm-year observations for 3,810 unique firms from 2000 through 2010.

### Measurement of Real Earnings Management

Following Roychowdhury (2006), we developed proxies for real earnings management. We consider the abnormal levels of cash flow from operations (CFO), discretionary expenses, and production costs to study the level of real activities manipulations.

The normal level of discretionary expenses (*DISC*) is estimated as a function of lagged sales based on a cross-sectional regression for the target firm’s industry each year:


(1) $$\frac{D\,I\,S\,C_{i.t}}{A\,s\,s\,e\,t_{i,t-1}}=\beta_{0}+\beta_{1}\,\frac{1}{A\,s\,s\,e\,t_{i,t-1}}+\beta_{2}\,\frac{S\,a\,l\,e\,s_{i,t-1}}{A\,s\,s\,e\,t_{i,t-1}}+\varepsilon_{i,t},$$

where *DISC* is defined as the sum of advertising expenses (COMPUSTAT annual data item #45), R&D (item #46), and selling, general, and administrative (SG&A) expenses (item #189).^2^ We scale all regression variables by total assets at the end of year *t*-1 and winsorize them at the 1st and 99th percentiles. The abnormal portion of *DISC* (*AB_DISC*) is, therefore, measured by the difference between the target firm’s actual *DISC* and its normal level of *DISC* as predicted by the solution to Equation (1).

Similarly, we estimate normal *PROD* level as a function of contemporaneous sales in absolute terms as well as both the contemporaneous change and the lagged change in sales using a cross-sectional regression for each industry and year:


(2) $$\frac{P\,R\,O\,D_{i.t}}{A\,s\,s\,e\,t_{i,t-1}}=\beta_{0}+\beta_{1}\,\frac{1}{A\,s\,s\,e\,t_{i,t-1}}+\beta_{2}\,\frac{S\,a\,l\,e\,s_{i,t}}{A\,s\,s\,e\,t_{i,t-1}}+\beta_{3}\,\frac{△\,S\,a\,l\,e\,s_{i,t}}{A\,s\,s\,e\,t_{i,t-1}}+\beta_{4}\,\frac{△\,S\,a\,l\,e\,s_{i,t-1}}{A\,s\,s\,e\,t_{i,t-1}}+\varepsilon_{i,t},$$

where *PROD* is defined as the sum of the costs of goods sold (COMPUSTAT annual data item #41) and changes in inventories (item #3). All the variables are scaled by total assets at the end of year *t*-1 and winsorized at the 1st and 99th percentiles. Again, an abnormal *PROD* level (*AB_PROD*) is computed as the gap between the actual *PROD* incurred and the firm’s normal *PROD* level, inferred by estimated coefficients from equation (2).

We express normal *CFO* as a linear function of sales and change in sales. To use this model, we run the following cross-sectional regression for each industry and year:


(3) $$\frac{C\,F\,O_{i,t}}{A\,s\,s\,e\,t\,s_{i,t-1}}=\beta_{0}\,\frac{1}{A\,s\,s\,e\,t\,s_{i,t-1}}+\beta_{1}\,\frac{S\,a\,l\,e\,s_{i,t}}{A\,s\,s\,e\,t\,s_{i,t-1}}+\beta_{2}\,\frac{\Delta \,S\,a\,l\,e\,s_{i,t}}{A\,s\,s\,e\,t\,s_{i,t-1}}+\varepsilon_{i,t}$$

where *CFO* is cash flow from operations in period *t* (Compustat data item #308–#124); we scale all regression variables by total assets at the end of year *t*-1 and winsorize them at the 1st and 99th percentiles. Abnormal *CFO* (*AB_CFO*) is the actual *CFO* minus the normal level of *CFO* calculated using the estimated coefficient from Equation (3).

We divided real earnings management into three measurements, following Cohen et al. (2008) and McGuire et al. (2012). The first, *REM1*, is the sum of the standardized two of abnormal discretionary expenditures (*AB_DISC*) and abnormal cash flows (*AB_CFO*). Second, *REM2* is the sum of the standardized three of abnormal discretionary expenditures (*AB_DISC*), abnormal production costs (AB_PROD), and abnormal cash flows (*AB_CFO*). The higher values for *REM1* and *REM2* represent higher levels of real earnings management.^3^

Table 1 presents summary statistics for the main variables in the analysis across all firm-years. The variables are defined in the Appendix. The average (median) value of our main explanatory variable, *Religion*, is 0.520 (0.507). The mean (median) *REM1* and *REM2* in our sample are −0.086 (−0.072) and −0.151 (−0.137), respectively. In addition, sample firms have a 5.1% *ROA*, a market-to-book ratio (*MKBK*) of 3.240, and a *Board Independency* of 74.9%, respectively. Overall, our summary statistics are similar in most important respects to those of samples used in prior studies (e.g., Coles et al., 2014).

**TABLE 1:** Descriptive statistics.

Variable	Mean	Q1	Median	Q3	SD
REM1	–0.086	–0.220	–0.072	0.040	0.234
REM2	–0.151	–0.384	–0.137	0.060	0.404
Religion	0.520	0.496	0.507	0.520	0.071
ROA	0.051	0.025	0.061	0.099	0.098
Size	7.758	6.684	7.621	8.664	1.464
Leverage	4.365	0.045	0.219	0.401	11.093
MKBK	3.240	1.583	2.425	3.772	3.458
Firm age	55.254	50.388	55.00	60.00	6.427
MTR	0.192	0.021	0.264	0.350	0.158
BIG4	0.954	1.000	1.000	1.000	0.210
Z-score	5.152	2.516	3.825	5.998	4.637
Board independency	0.749	0.667	0.778	0.875	0.138
Board size	2.161	1.946	2.197	2.398	0.275
IO	0.256	0.000	0.186	0.428	0.257
E-index	2.416	1.000	2.000	3.000	1.505
CEO age	4.009	3.928	4.007	4.094	0.111
CEO tenure	2.276	2.079	2.293	2.497	0.329
Duality CEO	0.273	0.000	0.000	1.000	0.445

*This table reports the summary statistics of all variables. The sample period is from 2000–2010, and all of the continuous variables are winsorized at the 1st and 99th percentiles to minimize the influence of outliers.*

## Empirical Results

### Effects of Religion on Real Earnings Management

One norm of religion that has been proved to significantly shape corporate behaviors is ethics. Religion provides adherents guidelines that help identify ethical and unethical experiences (Weaver and Agle, 2002). Therefore, individuals with religious beliefs consider involvement in unethical activities as unacceptable practices (Conroy and Emerson, 2004). Prior research has examined the influences of religion on ethical organizational decision-making. For example, Boone et al. (2013) show that firms in more religious counties are associated with fewer tax avoidance activities. Callen and Fang (2015) imply that religion helps mitigate bad news-hoarding activities by managers. The effects of religion on ethical firms’ decision-making also extend to financial reporting. McGuire et al. (2012) find that firms located in religious areas experience lower incidences of engaging in financial reporting irregularities. Building on previous findings and arguments, we expect a firm with higher religion will decrease earnings management activities. To test this conjecture, we estimate an OLS regression model as follows:


(4) $$R\,E\,M_{i,t}=\alpha_{0}+\alpha_{1}\,R\,e\,l\,i\,g\,i\,o\,n_{i,t-1}+\theta^{\prime }\,{\text{Z}}_{i,t-1}+\gamma_{i}+\mu_{t}+\varepsilon_{i,t},$$

where the dependent variables are *REM1*_*i,t*_ is the sum of the standardized two of abnormal discretionary expenditures (*AB_DISC*) and abnormal cash flows (*AB_CFO*). *REM2*_*i,t*_ is the sum of the standardized three of abnormal discretionary expenditures (*AB_DISC*), abnormal production costs (*AB_PROD*), and abnormal cash flows (*AB_CFO*). *Religion*_t–1_ as the number of religious adherents in the county to the total population in the county. *Z*_*i,t–1*_ is the vector of the control variables from the firm and corporate governance factor *I* in year *t-1*. γ_*i*_,μ_*t*_, and ε_*i*,*t*_ represent the firm and year fixed effects and the error of the regression. Control variables are defined in the Appendix and the continuous variables are winsorized at the 1st and 99th percentiles. Standard errors are clustered at the firm level.

Empirical evidence is presented in Table 2. Our results show that *Religion* significantly reduces earnings management, which are proxied by *REM1* and *REM2*. Results of earnings management that are measured by *REM1* are shown in columns (1–4) of Table 2. We find that estimated coefficients are significantly and negatively associated with *REM1* after considering firm-level characteristics and corporate governance factors. In addition, results are shown in columns (5) also imply that *Religion* imposes negative influences on earnings management after including both firm-level characteristics and corporate governance factors. These findings imply that local social norm does matter in firm decision-making, which is consistent with previous findings (Hilary and Hui, 2009; McGuire et al., 2012). Overall, we suggest that firms located in a higher level of religiosity mitigate the extent of earnings management.

**TABLE 2:** Religion and real earnings management (REM).

	(1)	(2)	(3)	(4)	(5)
	**REM1**	**REM1**	**REM1**	**REM1**	**REM2**
Religion	−0.754***	−0.858***	−0.740***	−0.824***	−1.245***
	(−3.45)	(−3.53)	(−3.08)	(−3.20)	(−3.26)
ROA		0.132***		0.123**	0.184*
		(2.76)		(2.28)	(1.72)
Size		0.043***		0.063***	0.099***
		(6.83)		(7.95)	(8.45)
Leverage		0.023		−0.021	−0.050
		(0.88)		(−0.67)	(−1.06)
MKBK		−0.007***		−0.004*	−0.007**
		(−3.88)		(−1.70)	(−2.11)
Firm age		−0.001		−0.004*	−0.005*
		(−0.47)		(−1.70)	(−1.74)
MTR		0.035**		0.046***	0.037
		(2.16)		(2.61)	(1.40)
BIG4		0.025		0.025	0.028
		(1.20)		(0.98)	(0.73)
Z-score		−0.006***		−0.007***	−0.012***
		(−3.78)		(−3.97)	(−4.48)
Board independency			−0.046**	−0.056**	−0.046*
			(−2.10)	(−2.12)	(−1.78)
Board size			0.003	−0.022	−0.046*
			(0.21)	(−1.24)	(−1.80)
IO			0.023	0.070**	0.098**
			(0.84)	(2.13)	(2.01)
E-index			−0.003	−0.003	−0.001
			(−0.92)	(−0.93)	(−0.29)
CEO age			0.031	0.036	0.095
			(0.83)	(0.86)	(1.56)
CEO tenure			−0.001	0.002	−0.003
			(−0.008)	(0.15)	(−0.17)
Duality CEO			0.004	−0.005	−0.024
			(0.66)	(−0.69)	(−0.71)
Year FE	Yes	Yes	Yes	Yes	Yes
Firm FE	Yes	Yes	Yes	Yes	Yes
Obs.	4,267	2,890	3,073	2,442	2,442
Adj. R-squared	0.876	0.893	0.885	0.9022	0.931

*This table reports the OLS regression results of religion on real earnings management. The empirical model is:*

$$R\,E\,M_{i,t}=\alpha_{0}+\alpha_{1}\,R\,e\,l\,i\,g\,i\,o\,n_{i,t-1}+\theta^{\prime }\,{\text{Z}}_{i,t-1}+\gamma_{i}+\mu_{t}+\varepsilon_{i,t},$$

where the dependent variables are REM1_i,t_ is the sum of the standardized two of abnormal discretionary expenditures (AB_DISC) and abnormal cash flows (AB_CFO). REM2_i,t_ is the sum of the standardized three of abnormal discretionary expenditures (AB_DISC), abnormal production costs (AB_PROD), and abnormal cash flows (AB_CFO). Religion_t–1_ as the number of religious adherents in the county to the total population in the county. Z_i,t–1_ is the vector of the control variables from the firm and corporate governance factor i in year t-1. γ_i_,μ_t_, and ε_i,t_ represent the firm and year fixed effects and the error of the regression. In all models, the t-values are computed on heteroskedasticity-robust standard errors (White, 1980). Coefficients: ***, **, and * denote significance at the 1, 5, and 10% levels, respectively.

### Endogeneity Problems

One problem that may appear is the omitted-variable bias due to the unobservable characteristics of tenure-weighted co-option. Lin et al. (2018) mention that the firm-fixed-effect model offers a solution for the unobservable omitted bias. In all regression estimates, we included the firm-fixed-effect to control for the unobservable omitted-variables bias. Furthermore, we carried out a natural experiment by using the regulation of the Sarbanes-Oxley Act (SOX) of 2002 as an exogenous shock of tenure-weighted religion and listing the requirements by NASDAQ and NYSE for firms to have a majority of independent directors. Following Coles et al. (2014), we modify the typical DID setup to isolate the effect of the religiosity level of firms’ location, which is termed as the “clean” effect. The main difference between the typical DID model and the modified DID setup is that it allows for the possibility that SOX has a direct effect on earnings management as well as an effect on the religiosity level where firms are located. This is because other regulations and political pressure arising from SOX were likely to decline earnings management through monitoring channels.

To assess the impact of local religious beliefs on firms’ earnings management, we estimate the modified DID model is shown below:


(5) $$R\,E\,M_{i,t}=\beta_{0}+\beta_{1}\,R\,e\,l\,i\,g\,i\,o\,n_{i,t-1}+\beta_{2}\,P\,o\,s\,t-S\,O\,X_{i,t}$$

$$\times R\,e\,l\,i\,g\,i\,o\,n_{i,t-1}+\beta_{3}\,N\,o\,n\,c\,o\,m\,p\,l\,i\,a\,n\,t_{i,t}\times R\,e\,l\,i\,g\,i\,o\,n_{i,t-1}$$

$$+\beta_{4}\,P\,o\,s\,t-S\,O\,X_{i,t}\times N\,o\,n\,c\,o\,m\,p\,l\,i\,a\,n\,t_{i,t}\times R\,e\,l\,i\,g\,i\,o\,n_{i,t-1}$$

$$+\beta_{5}\,P\,o\,s\,t-S\,O\,X_{i,t}+\beta_{6}\,N\,o\,n\,c\,o\,m\,p\,l\,i\,a\,n\,t_{i,t}$$

$$+k\,{\left(o\,t\,h\,e\,r\,c\,o\,n\,t\,r\,o\,l\,s\right)}_{i,t-1}+\varepsilon_{i,t},$$

where the dependent variables are *REM*_*i*,*t*_is the sum of the standardized abnormal discretionary expenditures (AB_DISC) and abnormal cash flows (AB_CFO). *REM2_*i*,t_* is the sum of three standardized variables: abnormal discretionary expenditures (AB_DISC), abnormal production costs (AB_PROD), and abnormal cash flows (AB_CFO). The measurement of our dependent variable in this modified DID setup follows previous papers (Cohen et al., 2008; McGuire et al., 2012), and is termed as *REM1* and *REM2*, respectively. Following Hilary and Hui (2009), *Religion*_*i*,*t*−1_ is the number of religious adherents in the county to the total population in the county. *Post*−*SOX*_*i*,*t*_is equal to one if the year is 2002 or later, and equal to zero otherwise. *Noncompliant*_*i*,*t*_is equal to one if the firm was not in compliance in 2001 and zero otherwise. *Other control* is the vector of the control variables from the firm and corporate governance factor. ν_*k*_,μ_*t*_,and ε_*i*,*t*_ represent the year fixed effect and the error of the regression.

The typical DiD contains three key dummy variables: *Post-SOX*, *Noncompliant*, and *Post-SOX* *Noncompliant*. To interpret the results of this modified DID model, we first focus on firms in the compliant pre-SOX group and firms in the compliant post-SOX group, where the response effects on earnings management are β_1_ and β_1_ + β_2_, respectively. We thereby obtain the effects of SOX is β_2_.

Our primary interest is the noncompliant post-SOX group. The response changes on earnings management in the firm for this group (= β_1_ + β_2_ + β_3_ + β_4_) are contaminated by the SOX effects other than the religiosity level that firms are located in and thus represent the combined effect of religious belief (clean effect) and SOX on the variable of interest. Therefore, the estimation of “clean” effect is given by β_1_ + β_3_ + β_4_ [(“clean” effect + SOX) – SOX].

Table 3 presents the DID clean estimate results for *religion* on real earnings management. We present the clean estimates for the impact of religiosity level that firms located on two measurements of real earnings management. We also report results from the baseline regressions for ease of comparison. The estimation of the “clean” effect on firms’ earnings management is negative and statistically significant (clean estimates are −0.561 and −0.824, respectively), which have the same sign and similar statistical significance relative to the baseline results (base case estimates are −0.824 and −1.245, respectively). The results reinforce our main finding that firms with higher *religion* are less likely to manipulate real earnings management.

**TABLE 3:** Difference-in-differences.

Coefficient estimate	Results from base case	“Clean” estimate
Table 2: Model 4	−0.824***	−0.561***
REM1	(−3.20)	(−2.62)
Table 2: Model 5	−1.245***	−0.824***
REM2	(−3.26)	(−2.59)

*This table presents the effect of religion on real earnings management using a natural experiment. The DiD model is:*

$$\begin{array}{l} REM_{i,t}={\text{β}}_{0}+{\text{β}}_{1}{\text{Religion}}_{i,t-1}+{\text{β}}_{2}Post-SOX+{\text{β}}_{i,t}\times {\text{Religion}}_{i,t-1}\\ +{\text{β}}_{3}Noncompliant+{\text{β}}_{i,t}\times {\text{Religion}}_{i,t-1}+{\text{β}}_{4}Post-SOX_{i,t}\\ \times Noncompliant+{\text{β}}_{i,t}\times {\text{Religion}}_{i,t-1}+{\text{β}}_{5}Post-SOX+{\text{β}}_{i,t}\\ +{\text{β}}_{6}Noncompliant_{i,t}+k{\left(other\;controls\right)}_{i,t-1}\varepsilon_{i,t}, \end{array}$$

where the dependent variables are REM1_i,t_ is the sum of the standardized two of abnormal discretionary expenditures (AB_DISC) and abnormal cash flows (AB_CFO). REM2_i,t_ is the sum of the standardized three of abnormal discretionary expenditures (AB_DISC), abnormal production costs (AB_PROD), and abnormal cash flows (AB_CFO). Religion_t–1_ as the number of religious adherents in the county to the total population in the county. Post-SOX_i,t_ is equal to one if the year is 2002 or later, and equal to zero otherwise. Noncompliant_i,t_ is equal to one if the firm was not in compliance in 2001 and zero otherwise. The typical DiD is the interaction term of Post−SOXxNoncompliantxReligion, but it does not yield the “Clean” estimate. The “clean” estimate of DiD is β_1_ + β_3_ + β_4_ = 0. Other control is the vector of the control variables from the firm and corporate governance factor. ν_k_,μ_t_, and ε_i,t_ represent the year fixed effect and the error of the regression. In all models, the t-values are computed on the heteroskedasticity-robust standard errors (White, 1980). Coefficients: ***, **, and * denote significance at the 1, 5, and 10% levels, respectively.

Another problem that may appear is self-selection bias. A corporate decision is usually deliberated on by managers, who make a selection according to their preferences. Even if we control the firm-fixed effect to reduce the omitted-variables bias, we cannot entirely reduce the self-selection bias problem. Following Rosenbaum and Rubin (1983) and Lin et al. (2018), we adopt a propensity-score-matching procedure to address the self-selection bias problem. We sort the level of *religion* into quartiles, and we define the top quartiles as the highest *religion* (treatment) and the bottom quartiles as the lowest *religion* (control). We confirm that the treatment group increases real earnings management compared to the control group. For a test of robustness, we used six different matching methods: (1) Near Neighbor (*n* = 1), (2) Near Neighbor (*n* = 2), (3) Near Neighbor (*n* = 3), (4) Kernel Gaussian, (5) Kernel Epanechnikov, and (6) Radius (1.0). Most of the results support our hypothesis.

Table 4 presents the average earnings management of both treatment firms and control firms and the difference in earnings management between groups. We find that the differences are negative and significant for both two measurements of earnings management. Compared to control firms, treatment firms are less likely to engage in earnings management activities. This result supports our view that a higher level of local religious beliefs negatively influences the practice of earnings management.

**TABLE 4:** A propensity-score-matching (PSM).

Matching method	Treatment	Control	Difference	*t*-Statistic
**Panel A: Real earnings management (*REM1*)**				
(1) Near neighbor (*n* = 1)	−0.085	−0.075	−0.010*	(−1.61)
(2) Mahalanobis	−0.085	−0.081	−0.040**	(−1.97)
(3) Kernel Epanechnikov	−0.085	−0.079	−0.060**	(−2.01)
**Panel B: Real earnings management (*REM2*)**				
(1) Near neighbor (*n* = 1)	−0.153	−0.139	−0.014*	(−1.79)
(2) Mahalanobis	−0.153	−0.145	−0.080**	(−2.12)
(3) Kernel Epanechnikov	−0.153	−0.140	−0.013**	(−2.06)

This table examines the effect of religion on real earnings management by using propensity-score-matching. We define the treatment group as those in the top quartile for Religion and the control group as those in the bottom quartile. Matching starts with firm characteristics. For robustness results, we used three different matching methods: (1) Near Neighbor (n = 1), (2) Mahalanobis, (3) Krenel Epanechnikov. In all models, the t-values are computed on the heteroskedasticity-robust standard errors (White, 1980). Coefficients: ***, **, and * denote significant at 1, 5, and 10% levels, respectively.

Overall, both methods for addressing the omitted-variable bias and the self-selection bias. Robustness results in this section confirm our main result that *religion* has a negative impact on real earnings management. Furthermore, our paper provides evidence that attributes to previous findings that local social norm does matter in firm decision-making (Hilary and Hui, 2009; McGuire et al., 2012).

## Conclusion

Using a sample of 3,810 unique firms in the U.S. from 2000 to 2010, we investigate whether local religious beliefs have a significant impact on the practice of earnings management. Our results imply that firms located in a U.S. County with high levels of religiosity are adopting restrained earnings management practices, which is consistent with the previous empirical findings that local social norms play an important role in corporate decision-making (Hilary and Hui, 2009; McGuire et al., 2012). Moreover, we mitigate the omitted-variable concern and self-selection concern by employing the difference-in-differences and Propensity score matching approaches, respectively, and find that our main results remain unchanged. As such, our work makes pivotal contributions to both the literature on the effects of religion on organizational behavior and determinants of earnings management activities. Furthermore, we confirm the significant negative relationship between local religiosity and earnings management, indicating that the local social environment that firms operate in is a crucial factor influencing earnings management levels. Finally, a key policy implication inspired by the result is that the effectiveness of local religious beliefs on firms’ behavior is affirmative, which is considered an important factor for firms’ governance.

## Data Availability Statement

Publicly available datasets were analyzed in this study. This data can be found here: http://www.thearda.com/.

## Author Contributions

All authors listed have made a substantial, direct, and intellectual contribution to the work, and approved it for publication.

## Conflict of Interest

The authors declare that the research was conducted in the absence of any commercial or financial relationships that could be construed as a potential conflict of interest.

## Publisher’s Note

All claims expressed in this article are solely those of the authors and do not necessarily represent those of their affiliated organizations, or those of the publisher, the editors and the reviewers. Any product that may be evaluated in this article, or claim that may be made by its manufacturer, is not guaranteed or endorsed by the publisher.

## Footnotes

1
http://www.thearda.com/

2 As long as there are available data on SG&A expenses, missing values for advertising or R&D expenses would be set to 0, since such expenses might have already been included in SG&A and hence not shown.

3 Following a prior paper, we multiply both abnormal discretionary expenses and abnormal cash flows by negative 1.

## Appendix

**APPENDIX TABLE 1:**

Variable	Definition	Data source
**Dependent variables**		
REM1	The sum of the standardized of abnormal discretionary expenditures (*AB_DISC*) and abnormal cash flows (*AB_CFO*)	Compustat
REM2	The sum of the standardized of abnormal discretionary expenditures (*AB_DISC*), abnormal production costs (*AB_PROD*), and abnormal cash flow (*AB_CFO*).	Compustat
**Independent variables**		
Religion	We calculate Religion as the number of religious adherents in the county to the total population in the county as reported by ARDA. Following previous studies (e.g., Alesina and La Ferrara, 2000; Hilary and Hui, 2009), we linearly interpolate the data to obtain the values for missing years (1972–1979, 1981–1989, and 1991–1999).	ARDA website
**Firm characteristics**		
ROA	The total net income divided by total assets	Compustat
Size	The natural logarithm of total assets	Compustat
Leverage	The total debt divided by total assets	CRSP
MKBK	The market value of equity divided by the book value of equity	Compustat
Firm age	The number of years since first listing in CRSP	CRSP
MTR	The tax rate on each additional dollar of income earned today	Graham and Mills (2008)
BIG4	A dummy variable equal to 1 if the firm is audited by top 5 auditors, and otherwise 0	I/B/E/S
Z-score	(1.2 × working capital + 1.4 × retained earnings + 3.3 × EBIT + 0.999 × sales)/total assets	Compustat
**Corporate governance characteristics**		
Board independency	The percentage of independent directors divided by board size	RiskMetrics
Board size	The natural logarithm of total board size	RiskMetrics
IO	The percentage of institutional ownership divided by total outstanding shares	Thomson-Reuters 13F
E-index	The entrenchment index based on six provisions	Bebchuk et al. (2009)
**CEO characteristics**		
CEO age	The natural logarithm of the CEO’s age	RiskMetrics
CEO tenure	The natural logarithm of the CEO’s tenure	RiskMetrics
Duality CEO	A dummy variable equal to 1 if the CEO is also the chairman, and otherwise 0	RiskMetrics
